# Gender Differences in Pulmonary Function, Respiratory Symptoms, and Macrophage Proteomics among HIV-Infected Smokers

**DOI:** 10.1155/2014/613689

**Published:** 2014-03-04

**Authors:** Shiva D. Rahmanian, Karen L. Wood, Shili Lin, Mark A. King, April Horne, Shangbin Yang, Haifeng M. Wu, Philip T. Diaz

**Affiliations:** ^1^Division of Pulmonary, Critical Care, and Sleep Medicine, Department of Medicine, The Ohio State University Medical Center, Columbus, OH, USA; ^2^201 Davis Heart and Lung Institute, 473 West 12th Avenue, Columbus, OH 43210, USA; ^3^Department of Statistics, The Ohio State University, Columbus, OH 43210, USA; ^4^Department of Radiology, The Ohio State University Medical Center, Columbus, OH 43210, USA; ^5^Department of Pathology, The Ohio State University Medical Center, Columbus, OH 43210, USA

## Abstract

*Background*. HIV-infected subjects have an increased incidence of pulmonary emphysema. There are known gender differences in COPD phenotypic expression and diagnosis, but this is not well characterized in lung disease related to HIV. We analyzed a group at risk for the development of COPD (HIV-infected smokers) to determine gender differences in pulmonary symptoms, pulmonary function tests, and HRCT appearances. *Methods*. This was a cross-sectional, baseline analysis of a prospective study performed between 2006 and 2010. We performed symptomatic, pulmonary function, and computed tomography assessments in 243 HIV-infected smokers. In a subset bronchoalveolar lavage was performed with proteomic analysis of their alveolar macrophages. *Results*. The majority of the participants were male 213 (87.6%). There was significantly higher percentage of cough and phlegm production in males. There was also a lower FEV1 and a higher RV in males than females. Proteomic analysis revealed 29 proteins with at least a 2-fold higher expression in males and 13 identified proteins that were higher in females. *Conclusions*. In this group of HIV-infected smokers, airway symptoms and pulmonary function test abnormalities were higher in men than women. These gender differences may be due to differential expression of certain proteins in this group.

## 1. Introduction

The prevalence of chronic obstructive pulmonary disease (COPD) is increasing dramatically in women. In fact, COPD now kills more women than breast and lung cancer combined [1] and the number of new cases of COPD is increasing three times as fast in women annually as compared to men [2]. Data suggests that there are differences in the presentation and phenotypic expression of COPD in women compared to men [3], While women may be more susceptible to early onset COPD, there appears to be a gender bias in the diagnosis of COPD, with women less likely to be diagnosed than men with similar symptoms [4, 5].

Human immunodeficiency virus (HIV) has emerged as an independent risk factor for COPD in smokers, as data in the pre- and postantiretroviral (ART) era demonstrate increased susceptibility to cigarette smoke and a high percentage develop abnormalities in lung function, including loss of diffusing capacity and irreversible air-flow obstruction [6, 7]. As ART has transformed HIV into a chronic disease, noninfectious pulmonary comorbidities are assuming greater importance for this population [8]. Whether cigarette smoking affects HIV-infected women differently than men has not been systematically studied.

The purpose of the current study was to compare respiratory symptoms, lung function, and results of high resolution computed tomography (HRCT) scanning among HIV-infected women and men. While previous studies have defined phenotypic differences between men and women in advanced COPD in the general population [4, 5], we were interested in studying an at risk group of smokers at an earlier disease stage. In addition, we examined alveolar macrophage proteomics in a subgroup of males and females. We wished to determine whether differences in protein expression existed between the two groups and whether such differences could provide mechanistic insight into observed symptomatic and physiologic differences.

## 2. Materials and Methods

This was a cross-sectional, baseline analysis of a prospective study performed between 2006 and 2010. The study involved the longitudinal assessment of the pulmonary status of HIV-infected subjects (*n* = 315) and included symptomatic, pulmonary function, and computed tomography assessment. In addition, a subset of agreeable subjects underwent bronchoalveolar lavage followed with proteomic analysis of their alveolar macrophages. The study was approved by the Ohio State University institutional review board (Biomedical Sciences IRB number 2005H0197) and all subjects signed informed consent. For the purposes of this analysis, only participants who had a history of cigarette smoking were included (*n* = 243).

### 2.1. Respiratory Symptoms

All subjects answered questions related to the presence or absence of respiratory symptoms, specifically, shortness of breath, cough, phlegm production, and wheezing.

### 2.2. Pulmonary Function Studies

All participants underwent complete pulmonary function testing, including spirometry, as well as measurement of lung volumes and carbon monoxide diffusing capacity according to American Thoracic Society guidelines. Predicted equations for spirometry were those of Goldman [9], lung volumes Crapo [10], and diffusing capacity Miller [11].

### 2.3. Computed Tomography of the Chest

All subjects underwent HRCT (high resolution computed tomography) of the chest. Scans were performed on a Siemens multislice CT scanner (16-slice, 20-slice open CT, or 64-slice), without IV contrast. Inspiratory and expiratory images were performed. All scans were read by an experienced chest radiologist. The presence or absence of emphysema (bullae, thin-walled cystic spaces, or abnormal decreases in attenuation accompanied by vascular disruption) was recorded, as was the presence of bronchial dilatation, bronchial wall thickening, and air trapping as previously described [12].

### 2.4. Alveolar Macrophage (AM) Proteomics

To examine alveolar macrophage proteomics, we matched 6 female subjects with 6 male subjects of similar age, smoking history, and use of ART. Briefly, a bronchoalveolar lavage (BAL) in the right middle lobe was performed to obtain a lavage sample of approximately 50 mL for the isolation of AMs [13]. After obtaining BAL, an initial centrifugation was performed to spin down AMs. AM purity in each cell preparation was evaluated by light microscopic examination of diff-quick cytospins to ensure at least 90% purity. After preparation of AMs, a small aliquot of AM cells was suspended in RIPA buffer for protein concentration measurement using BCA protein assay [14]. Afterwards, AMs obtained from each participant were lysed at a density of 3–5 × 10^6^ cells/mL in 2D gel cell lysis buffer [13] and frozen at –80°C for 2D gel proteomic analysis performed in batches.

For first dimension electrophoresis, 100 *μ*L cell lysates (~2 mg cellular proteins) were mixed with 400 mL rehydration buffer and spun at 14,000 g, at 4°C for 10 minutes. Four hundred fifty *μ*L of the supernatant was then subjected to first D electrophoresis overnight on an Amersham IPGphor using premade 24 cm IPG strips. Following first D electrophoresis, the strip was equilibrated in a buffer containing for 10 min. at room temperature. This is followed by a second equilibration for 10 min. Second dimension electrophoresis was run on a 20 × 24 cm SDS-PAGE on an Amersham Dalt II, a large format (20 × 24 cm) 2D gel system. Under this condition, a total of approximately 1,100–1,500 protein spots were detected and analyzed in each AM sample.

After 2D electrophoresis, the gels were fixed and stained with SyproRuby fluorescence dye according to manufacturer's protocol. Gel images were captured on a Typhoon 9200 laser scanner (Amersham) that offers high resolution and quantification of protein spots. Protein quantification on all 2D gels was performed using ImageMaster 2D software (Nonlinear Dynamics) [13, 15, 16]. Proteins demonstrating significant and reproducible differences between male and female HIV smokers were subjected to protein identity determination using tandem mass spectrometry at OSU's Proteomic Shared Core Facility.

### 2.5. Statistical Analysis

The prevalence of respiratory symptoms, findings on high resolution chest CT, and pulmonary function testing were analyzed using linear/logistic models with sex, age, pack-years smoking history, viral load, BMI, and IV drug use as covariates. Because of skewness in the data, log transformation was performed on pack-years smoking history, residual volume, BMI, and viral load before model fitting.

## 3. Results

### 3.1. Participants

Table 1 demonstrates the baseline characteristics of the study population. The sex distribution and demographics of the population were similar to that of the HIV demographics of Central Ohio 30 females (12.3%) and 213 males (87.6%). The mean age of the participants was 44.3 with 57.6% Caucasians, 40.7% African Americans, and 1.7% other. The average pack-year history of the group was 19; 63% of females and 65% of males were current smokers. The average viral load was 36,552 with an average CD4 count of 474.

### 3.2. Respiratory Symptoms, Pulmonary Function Testing, and Radiographic Imaging

Table 2 demonstrates clinical pulmonary findings among the subjects according to participant gender. Males had a significantly higher prevalence of cough (72.8% versus 51.7% (*P* = 0.0015)) and phlegm production (71.2% versus 51.7% (*P* = 0.025)) compared to females. There was no statistically significant differences between males and females with regard to shortness of breath (32.4% males versus 44.8% females (*P* = 0.87)) and wheezing (49.8% males versus 55.2% females (*P* = 0.89)).

A comparison of the HRCT findings did not reveal any differences regarding the prevalence of emphysema, bronchial dilatation, or bronchial wall thickening. Although there was no statistically significant difference in the prevalence of air trapping, there was a trend towards significance (29.1% males versus 22.2% females (*P* = 0.083)).

Based on pulmonary function testing, males had a lower percent predicted forced expiratory volume in one second (FEV1). The average FEV1 percent predicted was 91.4% in males versus 97.3% in females (*P* = 0.0086). There was also a higher prevalence of air trapping as measured by the residual volume (RV) in males compared to females with an average RV of 115.1% in males compared to 99.41% in females (*P* = 0.0496). While there was no significant difference between males and females with regard to diffusion impairment, both groups exhibited diffusion impairment with a mean diffusing capacity for carbon monoxide (DLCO) of less than 80% predicted in both groups (79.9% in males and 75.5% in females).

### 3.3. Proteomic Analysis

Table 3 demonstrates the subset of subjects that underwent BAL and proteomic analysis. There were 6 men and 6 women who were matched for HAART therapy, smoking status and pack years, age and race. Tables 4 and 5 demonstrate results of 2D gel alveolar macrophage proteomic analysis between men and women. Sixty-five proteins were identified that were at least twofold greater in men of which 29 were identified (Table 4). Thirty-eight proteins were identified that were at least twofold greater in women of which 13 were identified (Table 5).

## 4. Discussion

The current study indicates that in a population of HIV-infected smokers there are gender differences in pulmonary function and respiratory symptoms. Males have an increased prevalence of cough and phlegm production, as well as lower % of predicted FEV1 and a higher % of predicted RV. HRCT scanning demonstrated a trend to increased air trapping. Of note, the degree of diffusion impairment was similar between the two sexes as was the degree of emphysema on chest CT. The overall findings suggest among the HIV-infected population, male smokers may be more likely to develop early airways dysfunction than female smokers.

Studies examining gender differences among individuals with COPD in the general population suggest that women may be more susceptible to the effects of cigarette smoke. In a study by Silverman and colleagues [5] gender differences in severe early-onset COPD were studied by examining 84 early-onset COPD subjects and 348 of their first degree relatives. They found a similar level of airflow obstruction in male and female subjects; however, females had a tendency to smoke less. In first degree relatives, when analyzing current or exsmokers, female first degree relatives had a significantly lower FEV1/FVC ratio, significantly greater bronchodilator response, and more likely to have an FEV1% predicted less than 40%. It has been suggested that women may have a specific phenotype of COPD, that is, more airway disease predominant, whereas men tend to have more of an emphysematous phenotype. Based on a retrospective review of 1438 patients with a diagnosis of COPD, spirometric evidence of airflow obstruction, and CT scan data, a significantly greater proportion of women had airway disease [3]. Sex differences may exist on computed tomography as well, as it appears that men may have more emphysema on CT than women for the same degree of obstruction [17].

Our study is somewhat different from other studies that have examined established, advanced COPD; instead, we studied a group of at risk smokers finding that airway symptoms and loss of FEV1 appear to be more common in men. While airways disease seemed to be less affected in our population of women, diffusion impairment was prominent. Interestingly other studies on the non-HIV infected population have suggested that early in the presentation of COPD, women may have less airway disease, but greater diffusion impairment [18]. Furthermore, phlegm production has been seen to be more prominent in men with early COPD and respiratory symptoms better correlate with abnormal spirometry in men compared to women [19]. The different phenotypic expression of COPD at an early stage may in part explain why women are diagnosed at a later stage. Processes affecting gas exchange may be more clinically subtle and may not point to COPD as compared to airways disease. It should also be noted that the gender differences we have found may involve pathogenic factors unique to HIV and may not translate to the general population of smokers.

Our results differ from those of Gingo and colleagues who have recently reported lung function and respiratory symptoms among a cohort of HIV-infected subjects [7]. While both the diffusion impairment and respiratory symptoms were prominent in their population, the investigators did not report sex differences. Notably the populations are somewhat different as Gingo and colleagues included both smokers and never-smokers, while our analyses were confined smokers (either current or former). Furthermore, Gingo and coworkers grouped respiratory symptoms in their analyses, possible missing differences among types of symptoms (i.e., airway symptoms versus dyspnea) [7].

A limitation of our study was the disproportionate number of males compared to females, a reflection of the HIV-population in Central Ohio. Future studies, such as the ongoing multicenter Lung HIV study, are prospectively investigating lung function, and respiratory symptoms in over 4000 subjects [8]. Results of this investigation should provide additional insight into potential differences in respiratory outcomes among male and female smokers with HIV.

In addition to symptoms, PFTs, and radiographic difference, we performed alveolar macrophage proteomics in a subgroup of matched females and males to explore potential mechanisms underlying the phenotypic differences in this population. A number of proteins were significantly different between the two sexes. This hypothesis generating approach involves the investigation of the protein content of a biological system [20, 21]. Since biological phenotypes are largely determined by proteins, differential expression of certain proteins may have mechanistic implications with regard to the gender differences we have identified. These data are limited by the small number of subjects that were able to have bronchoscopy and alveolar macrophage proteomics. However, in this subset of patients, cathepsin D propreprotein and procathepsin H (both lysosomal proteins) are expressed at higher levels in the male group. It has been shown that Cathepsin D contributes to the multiple disease processes including breast cancer [22], Alzheimer disease [23], and HIV [24]. For example, Cathepsin D can modify the conformation of HIV-1 gp120 that in turn directly interacts with the CXCR4 coreceptor and thus enhances HIV infectivity by promoting the entry of HIV into cells [24]. Conceivably, Cathepsin D may contribute to the earlier manifestation of airway symptoms among the male HIV-infected smokers. Interestingly, cathepsin H has been shown to be upregulated in an IL-13 induced emphysema murine model [25]. Cathepsin H has also been shown to have a role in surfactant generation [26]. Another study showed that IFN gamma decreased the levels of surfactant protein B and it was thought to be through reduction in cathepsin H [27]. SP-B may be important in COPD susceptibility and frequency of exacerbations [28]. It is not clear what the cause or consequence of elevated procathepsin H levels in AM from HIV-infected men, but it is tempting to think it may play a role in the difference in symptoms between men and women. Cleary, these proteomic studies are “hypothesis generating” and follow-up studies are needed to further investigate these observations.

A recent study analyzed gender differences in the proteome of BAL cells in healthy smokers and subjects with COPD. They compared healthy nonsmokers, healthy smokers, and subjects with COPD, while we compared HIV-infected smokers. However, there are some similarities with both studies finding dysregulation of lysosomal proteins in females. In their cohort there was downregulation of cathepsin B in female subjects with COPD as compared with healthy smokers [29].

In conclusion, among a group of relatively young HIV-infected smokers there appear to be differences in the manifestation of respiratory abnormalities, as men seem to develop airway symptoms earlier than women, while both groups have prominent abnormalities in gas exchange. Alveolar macrophage proteomics demonstrate differences in protein expression between the two groups which may provide mechanistic insight. Future study into this phenomenon is warranted as this process may provide insight into COPD pathogenesis in the general population.

## Figures and Tables

**Table 1:** Baseline demographics of the study participants.

Demographics	All subjects
Subject number	243
Male number (%)	213 (87.6%)
Female number (%)	30 (12.3%)
Age, mean (std dev)	44.3 (8.4)
BMI, mean (std dev)	26.98 (8.35)
Race	
Caucasian (%)	40.7
African American (%)	57.6
Other (%)	1.7
IV drug use number (%)	43 (17.7%)
Current smokers number (%)	157 (64.6%)
Pack years, mean (std dev)	19 ± 20.1
CD4 count, mean (std dev)	473.85 ± 284.80
Viral load, mean (std dev)	36552.43 ± 118323.6

**Table 2:** Comparison of sex differences in respiratory symptoms, chest CT findings, and pulmonary function testing.

	Male	Female	Odds ratio/regression coefficient	*P* value
Symptoms				
Cough (%)	72.8	51.7	4.33	0.0015
Phlegm (%)	71.2	51.7	2.68	0.025
Shortness of breath (%)	32.4	44.8	1.08	0.87
Wheeze (%)	49.8	55.2	1.06	0.89
CT chest findings				
Emphysema (%)	45.8	33.3	1.51	0.39
Bronchial dilatation (%)	22.2	16.6	1.21	0.73
Air trapping (%)	29.1	22.2	2.62	0.083
Bronchial wall thickening (%)	16.5	13.3	1.11	0.86
Pulmonary function testing				
FEV1/FVC	76.3	78.3	−1.48	0.40
FEV1% predicted	91.4	97.3	−8.35	0.0086
RV% predicted	115.1	99.4	0.116*	0.0496
TLC% predicted	103.5	99.8	2.57	0.42
DLCO% predicted	79.9	75.5	4.35	0.19

*The regression coefficient for RV% predicted is in the log scale.

**Table 3:** Clinical characteristics of subjects undergoing bronchoalveolar lavage with alveolar macrophage proteomic analysis.

Gender	HAART	Current smoker	Pack years	Age	Race
F	Y	N	1	37	W
F	Y	Y	31	47	W
F	Y	Y	16	48	B
F	Y	Y	6	47	B
F	Y	Y	11	33	W
F	Y	Y	28	52	W
M	Y	N	1	50	W
M	Y	Y	36	42	W
M	Y	Y	11	44	B
M	Y	Y	11	51	B
M	Y	Y	14	34	W
M	Y	Y	29	45	W

**Table 4:** Proteins that had at least a 2-fold higher expression in alveolar macrophages from men.

Spot number	Mean of normalized volume of male	Mean of normalized volume of female	Fold changes (male/female)	Accession number (Swiss-Prot)	Protein ID	Mowse score	Observed pI/Mr (KDr)	Number of peptides by MS/MS	Total seq. coverage (%)
867	0.04867	0.02083	2.34	NP_006079	Tubulin, beta, 2	2103	5.0/53.3	8	62
1009	0.05650	0.02167	2.61	BAD96752	Beta-actin variant	471	7.5/45.1	8	31
1318	0.12600	0.02983	4.22	P11177	Pyruvate dehydrogenase E1 component subunit beta, mitochondrial	427	6.2/39.6	4	39
1773	0.22083	0.06217	3.55	NP_001900	Cathepsin D preproprotein	898	5.2/27.7	5	33
1802	0.06200	0.03067	2.02	P30041	Peroxiredoxin 6	364	6.0/27.4	7	76
1872	0.05017	0.01600	3.14	P25787	Proteasome subunit alpha type 2	265	6.6/25.7	6	76
3074	0.17217	0.07350	2.34	1IVY_A	Chain A, Physiological Dimer Hpp Precursor	230	4.5/29.4	3	9
3157	0.06600	0.03250	2.03	P05092	Cyclophilin A	206	6.4/16.4	3	52
3235	0.11967	0.01050	11.40	P11021	78 kDa glucose-regulated protein	2193	5.3/74.9	24	50
4880	0.09550	0.00567	16.85	CAA68491	Glutathione peroxidase	1143	5.5/23.6	4	89
4925	0.12867	0.00100	128.67	P26447	Protein S100-A4	208	5.9/11.9	5	45
4990	0.11867	0.01933	6.14	P00558	Phosphoglycerate kinase 1	2011	7.9/44.9	13	72
5173	0.11133	0.03450	3.23	P30048	Thioredoxin-dependent peroxide reductase, mitochondrial	204	7.7/28.0	3	16
5180	0.10050	0.03300	3.05	Q06830	Peroxiredoxin-1	636	7.3/22.2	7	47
8849	0.06850	0.00100	68.50	P54727	UV excision repair protein RAD23 homolog B	643	4.8/43.2	8	41
8872	0.05933	0.02533	2.34	P06576	ATP synthase subunit beta, mitochondrial	513	5.3/56.5	8	34
8985	0.09233	0.01900	4.86	P06733	Enolase 1	638	5.7/42.3	8	28
9003	0.15800	0.03033	5.21	AAA51580	Gamma-actin	620	4.6/43.3	3	19
9023	0.63483	0.28000	2.27	Q3ZCM7	Tubulin beta-8 chain	344	4.8/49.7	4	18
9103	0.05133	0.01850	2.77	P06396	Gelsolin	1241	5.9/85.6	4	25
9126	0.06717	0.01100	6.11	O95336	6-phosphogluconolactonase	331	5.7/27.8	4	39
9141	0.10867	0.03400	3.20	1HJO_A	Chain A, Heat-Shock 70 kd Protein 42 kd Atpase N-Terminal Domain	1329	7.4/28.6	5	45
9152	0.08583	0.00217	39.62	P09668	Procathepsin H	517	8.4/37.4	3	29
9153	0.13433	0.01400	9.60	P52566	Rho GDP-dissociation inhibitor 2	616	5.1/23.0	10	75
9169	0.09733	0.04633	2.10	AAA35607	IgE-binding protein	257	7.4/26.1	2	22
9337	0.10317	0.04183	2.47	1GLO_A	Chain A, Crystal Structure Of Cys25ser Mutant Of Human Cathepsin S	193	6.6/16.5	5	32
9408	0.29833	0.12683	2.35	1AWI_A	Chain A, Human Platelet Profilin complexed with The L-Pro10 Peptide	988	9.1/14.5	2	93
9504	2.49950	1.10067	2.27	P08670	Vimentin	2541	5.1/53.6	12	63
9734	0.11817	0.00100	118.17	P10809	60 kDa heat shock protein, mitochondrial	2283	5.7/61.0	13	56

Only identified proteins are shown. Accession number: international database of the name and ID number of protein. Fold change: average density of the specific protein in males compared to females. Mowse score: measures reliability of the protein identification; greater than 100 ensures higher reliability that the protein identified is correct. Observed pI/Mr: isoelectric point and molecular weight of the protein. Ms/Ms: number of peptides sequenced after digestion. Total sequence coverage: percentage of the peptide sequenced.

**Table 5:** Proteins that had at least a 2-fold higher expression in alveolar macrophages from women.

Spot number	Mean of normalized volume of male	Mean of normalized volume of female	Fold changes (female/male)	Accession number (Swiss-Prot)	Protein ID	Mowse score	Observed pI/Mr (KDr)	Number of peptides by MS/MS	Total seq. coverage (%)
778	0.0317	0.1257	3.97	P19971	Thymidine phosphorylase	326	5.2/55.0	8	31
947	0.4037	0.9677	2.40	O14773	Tripeptidyl-peptidase I	427	5.7/47.6	3	19
1526	0.0402	0.0855	2.13	P07355	Annexin A2	422	7.6/38.6	6	36
2342	0.0182	0.0443	2.44	Q16781	Ubiquitin-conjugating enzyme E2 N	134	5.7/15.7	2	22
2536	0.0575	0.1312	2.28	P01884	Beta-2-microglobulin	107	6.0/12.0	2	34
4917	0.2527	1.0122	4.01	CAA23759	Unnamed protein product	1004	7.0/13.6	2	95
4947	0.0657	0.1440	2.19	NP_004842	Napsin A preproprotein	1678	5.3/35.0	3	22
8844	0.0028	0.0513	18.12	Q02818	Nucleobindin-1	282	5.2/53.8	11	29
9158	0.0893	0.1852	2.07	1JHW_A	Chain A, Ca2+-binding mimicry in the crystal structure of the Eu3+-bound mutant human macrophage capping protein cap G	1063	4.9/26.3	5	34
9207	0.0112	0.0510	4.57	P07203	Glutathione peroxidase	320	5.5/24.2	4	54
9501	0.0447	0.0922	2.06	P43686-2	Proteasome 26S ATPase subunit 4 isoform 2	192	5.0/62.7	3	18
9629	0.0725	0.2178	3.00	P07237	Protein disulfide-isomerase	937	4.8/57.1	8	34
9658	0.0245	0.1052	4.29	P26038	Moesin	539	6.1/67.9	6	13

Only identified proteins are shown. Accession number: international database of the name and ID number of protein. Fold change: average density of the specific protein in males compared to females. Mowse score: measures reliability of the protein identification; greater than 100 ensures higher reliability that the protein identified is correct. Observed pI/Mr: isoelectric point and molecular weight of the protein. Ms/Ms: number of peptides sequenced after digestion. Total sequence coverage: percentage of the peptide sequenced.

## Abbreviations

COPD: Chronic obstructive pulmonary disease
ART: Antiretroviral
HRCT: High resolution computed tomography
AM: Alveolar macrophage
BAL: Bronchoalveolar lavage
FEV1: Forced expiratory volume in one second
RV: Residual volume
DLCO: Diffusing capacity for carbon monoxide
HIV: Human immunodeficiency virus.
